# Millets for a sustainable future

**DOI:** 10.1093/jxb/erae507

**Published:** 2024-12-26

**Authors:** Arindam Ghatak, Iro Pierides, Roshan Kumar Singh, Rakesh K Srivastava, Rajeev K Varshney, Manoj Prasad, Palak Chaturvedi, Wolfram Weckwerth

**Affiliations:** Molecular Systems Biology Lab (MOSYS), Department of Functional and Evolutionary Ecology, University of Vienna, Djerassiplatz 1, 1030 Vienna, Austria; Vienna Metabolomics Center (VIME), University of Vienna, Djerassiplatz 1, 1030 Vienna, Austria; Molecular Systems Biology Lab (MOSYS), Department of Functional and Evolutionary Ecology, University of Vienna, Djerassiplatz 1, 1030 Vienna, Austria; Department of Botany, Mahishadal Raj College, Purba Medinipur, Garh Kamalpur, West Bengal 721628, India; Center of Excellence in Genomics & Systems Biology, International Crops Research Institute for the Semi-Arid Tropics (ICRISAT), Hyderabad, India; WA State Agricultural Biotechnology Centre, Centre for Crop & Food Innovation, Food Futures Institute, Murdoch University, Murdoch, 6150, Western Australia, Australia; National Institute of Plant Genome Research (NIPGR), Aruna Asaf Ali Marg, New Delhi 110067, India; Department of Genetics, University of Delhi, South Campus, Benito-Juarez Road, New Delhi 110021, India; Molecular Systems Biology Lab (MOSYS), Department of Functional and Evolutionary Ecology, University of Vienna, Djerassiplatz 1, 1030 Vienna, Austria; Molecular Systems Biology Lab (MOSYS), Department of Functional and Evolutionary Ecology, University of Vienna, Djerassiplatz 1, 1030 Vienna, Austria; Vienna Metabolomics Center (VIME), University of Vienna, Djerassiplatz 1, 1030 Vienna, Austria; The Quadram Institute, UK

**Keywords:** Breeding, climate resilience, integrated omics, millets, multiomics, PANOMICS, stress tolerance, sustainable development goals

## Abstract

Our current agricultural system faces a perfect storm—climate change, a burgeoning population, and unpredictable outbreaks such as COVID-19 which disrupt food production, particularly for vulnerable populations in developing countries. A paradigm shift in agriculture practices is needed to tackle these issues. One solution is the diversification of crop production. While ~56% of the plant-based protein stems from three major cereal crops (rice, wheat, and maize), underutilized crops such as millets, legumes, and other cereals are highly neglected by farmers and the research community. Millets are one of the most ancient and versatile orphan crops with attributes such as fast growing, high yielding, withstanding harsh environments, and rich in micronutrients such as iron and zinc, making them appealing to achieve agronomic sustainability. Here, we highlight the contribution of millet to agriculture and focus on the genetic diversity of millet, genomic resources, and next-generation omics and their applications under various stress conditions. Additionally, integrative omics technologies could identify and develop millets with desirable phenotypes having high agronomic value and mitigating climate change. We emphasize that biotechnological interventions, such as genome-wide association, genomic selection, genome editing, and artificial intelligence/machine learning, can improve and breed millets more effectively.

## Teaser

This review outlines the use of marker-assisted breeding to develop a high-yielding and nutritious elite millet germplasm using advanced genomics tools, omics techniques, and systems biology approaches.

## Introduction

Climate change and the rise in the global population pose a severe threat to food security. Globally, to meet the demands of a rapidly increasing population, which is projected to reach 9–10 billion by 2050, there is a pressing need to boost food production by 60–110% while simultaneously addressing the alarming climatic fluctuations which are complex and unpredictable (Rockström *et al.*, 2017). The limited variety of crops supplying global food makes our agricultural system more susceptible to climate hazards. Currently, >50% of consumed calories come from just three staple crops (rice, maize, and wheat), neglecting the wide variety of nutrient-rich plants historically utilized by humanity (Hunter *et al.*, 2019). Expanding our knowledge about underutilized and neglected food crops that can provide nutrition and support sustainable agriculture is essential.

Millets collectively form a group of small-grained cereals that are broadly classified into major and minor millets; major millets include pearl millet (*Cenchrus americanus*), finger millet (*Eleusine coracana*), and sorghum [*Sorghum bicolor* (L.) Moench], whereas minor millets consist of foxtail millet (*Setaria italica*), little millet (*Panicum sumatrense*), proso (broomcorn) millet (*Panicum miliaceum*), kodo millet (*Paspalum scrobiculatum*), barnyard millet (*Echinochloa esculenta*), tef (*Eragrostis tef*), guinea millet (*Brachiaria deflexa*), fonio (*Digitaria exilis*), browntop millet (*Urochloa ramosa*), and Job’s tears (*Coix lacryma-jobi*) (Ajeesh Krishna *et al.*, 2022; Antony Ceasar and Maharajan, 2022). Millets are the oldest ancient crops, cultivated >8000 years ago. Millets have C_4_ photosynthetic physiology and belong to a family of the *Poaceae* and subfamily *Panicoideae* and *Chloridoideae*, which enables them to thrive under harsh climatic conditions. For instance, sorghum outperforms other cereals in rainfed and drought conditions (Chaturvedi *et al.*, 2022; Banshidhar *et al.*, 2023). Similarly, pearl millet, foxtail millet, proso millet, and kodo millet are well suited to extreme drought, high temperatures, low soil fertility, salinity, and acidic soils (Das and Rakshit, 2016; Ghatak *et al.*, 2017). They also demonstrate better water use efficiency, nitrogen use efficiency (NUE) (see Box 1), a short life cycle, and require fewer agricultural inputs compared with other popular cereal crops, prompting farmers to reintroduce millet cultivation and bring them back to the market (Satyavathi *et al.*, 2021).

Box 1. **Nitrogen use efficiency in millets**

C_4_ plant physiology renders millets with higher NUE due to their compartmentalized CO_2_-concentrating mechanism around Rubisco, a major N-storing enzyme crucial for photosynthesis (Evans and von Caemmerer, 1996). Dissection and integration of complex traits such as NUE requires a transition from a gene-centric view of crop bioengineering to a PANOMICS perspective (Weckwerth *et al.*, 2020; Ghatak *et al.*, 2023). Genetic engineering efforts identified the Dof1 transcription factor (TF) (Gupta *et al.*, 2014) and the PII signalling superfamily (Hsieh *et al.*, 1998) as potential candidates for NUE in millets. Recently, Bandyopadhyay and co-workers found potential regulation of NUE in contrasting N-responsive pearl millet genotypes by APETALA2 (AP2) TFs (Bandyopadhyay *et al.*, 2024). By the use of ^15^N/^13^C isotope labeling, the N-responsive genotype allocated more biomass to nodes and roots, more N to grains, and more effective N remobilization in the flag leaf (Bandyopadhyay *et al.*, 2024). Furthermore, soil microbes and their interactions with host plant species mediated by root exudates influence the N cycle in the rhizosphere (Ghatak *et al.*, 2023). Various symbionts release secondary metabolites that induce N-fixing bacteria to enter roots. Biological nitrification inhibition (BNI) by plant exudates reduces microbe nitrification, release of N_2_O and potentially increase NUE. Ghatak and co-workers detected enhanced BNI in contrasting drought-stressed pearl millet genotypes by control of microbial community activity through altered root exudation (Ghatak *et al.*, 2022). Wang and co-workers identified genome-wide associations of 827 foxtail millet cultivars to rhizoplane microbiota composition by a combined GWAS, microbiome-wide association studies (MWAS), and microbiome genome-wide association studies (mGWAS) analysis. Their analysis unraveled SNP-associated rhizoplane operational taxonomic units (OTUs) that had high correlations with plant growth traits, suggesting a genotype-dependent influence of root microbiome composition that affects agronomic traits of foxtail millet (Wang *et al.*, 2022). This methodology can also be applied to find key millet genetic variants that influence mircobiome composition with downstream effects on NUE. Advanced machine learning methods have been used to test the causal effect of selected genomic variants on NUE in Arabidopsis and in maize (Cheng *et al.*, 2021), and should also be applied in millets as a genome-wide pre-selection step before functional validation using CRISPR/Cas9.

Although millets are less popular than major cereal crops, they are highly nutritious due to their rich content of proteins, minerals, flavonoids, polyphenols, and vitamins, offering multiple health benefits (such as being gluten-free and having a low glycemic index), and have a long shelf life (Muthamilarasan and Prasad, 2021; Chaturvedi *et al.*, 2022, 2023; Kudapa *et al.*, 2023). Recognizing their nutritional and ecological advantages, the United Nations (UN) designated the year 2023 as the International Year of Millets (IYM; https://www.fao.org/millets-2023/en) to raise awareness and promote the cultivation of millets, supporting the UN’s sustainable development goals, particularly the goal of ‘attaining zero hunger’ (Goal 2) (UN General Assembly; https://www.un.org/sustainabledevelopment/hunger/). Despite their climate resilience, millet production suffers from issues related to domestication, such as seed shattering, low yield, lodging, and poor agronomic practices. These unfavorable traits have hindered their globalization.

**Fig. 1.**
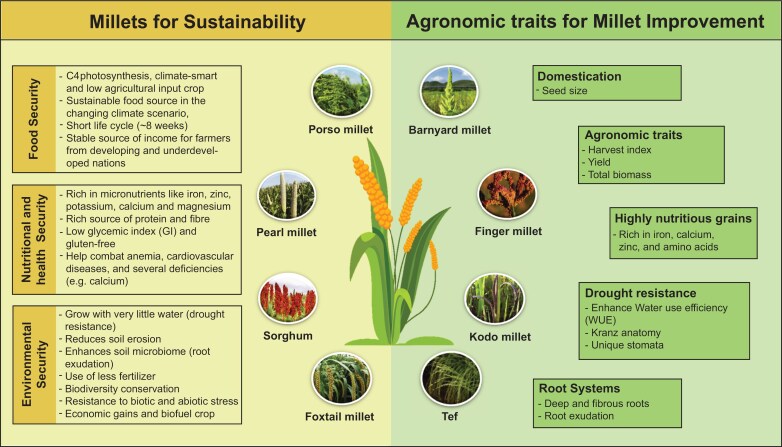
Millets for sustainable agriculture and relevant traits for target improvement.

However, the broad resilience characteristics of millets will be difficult to reproduce in other cereals because of the intricate genetic basis of abiotic stress tolerance. Hence, efforts should focus on improving yield traits in millets and shifting global production toward these important underutilized crops. Furthermore, understanding the unique traits of millet and their full potential in terms of productivity and climate resilience is still in its early stages due to the lack of well-characterized molecular information, which has limited yield improvement (Fig. 1). With the recent development of genomic information and advances in omics tools, there is potential to improve millets through marker-assisted breeding and exploiting trait-specific variations, leading to the expansion of existing germplasm (Weckwerth *et al.* 2020). We can use data-driven crop improvement to explore and combine useful variations to target specific traits and produce high nutrient value cultivars. This approach can also shorten the selection cycle and reduce the time to release new varieties, particularly important in changing climatic conditions. This review provides a comprehensive overview of modern genome sequencing in millet and summarizes the efforts undertaken for millet improvement using high-throughput molecular analysis and genotyping technologies.

## Genomic interventions for millet improvement

Significant advances have been made in developing climate-resilient crops by improving grain yields in major cereals such as rice, maize, and wheat. A pioneering effort to generate necessary genomic resources for enhancing breeding programs in pearl millet led to the formation of the international consortium that includes various organizations such as CGIAR, ICRISAT, and others, resulting in the assembly of the reference genome of the inbred line, Tift23D2B1-P1-P5, the first draft genome for pearl millet (Varshney *et al.*, 2017). This analysis resulted in the annotation of 27 893 (72.30%) protein-coding genes, with the expansion in gene families associated with terpenoid biosynthesis, which might explain the high heat and drought tolerance level in pearl millet compared with other cereals. The Tift 23D2B1-P1-P5 (BioSample identifier: SAMN04124419) pearl millet reference assembly is available in the NCBI (ASM217483v1) and the European Nucleotide Archive (GCA_002174835.1). More advanced, long-read sequencing was recently performed to improve the quality of the Tift 23D2B1-P1-P5 genome by generating Bionano Genomics optical maps and long reads obtained by Oxford Nanopore Technologies (ONT) sequencing (Salson *et al.*, 2023). This resulted in better continuity in the order of the contigs and the scaffolds of the new assembly, especially in the centromeric regions (Salson *et al.*, 2023), compared with bacterial artificial chromosome (BAC) sequencing. The genome sequences of other millets are also available, namely foxtail millet (Bennetzen *et al.*, 2012), tef (Cannarozzi *et al.*, 2014; VanBuren *et al.*, 2020), finger millet (Hittalmani *et al.*, 2017), and proso millet (broomcorn) (Zou *et al.*, 2019), further expanding the available resources for genetic improvement of various millets and enabling their use as model crop plants (see Box 2).

Box 2. **Key developments in understanding millet research for better adaptation to climate change**

He *et al.* (2023a) provided the first graph-based pan-genome of foxtail millet (*Setaria italica*), consisting of 110 diverse *Setaria* accessions (35 wild, 40 landraces, and 35 modern cultivars). Large-scale genetic studies across 13 different environments were performed, which can be crucial for accelerating marker-assisted breeding, gene editing, and developing improved breeding programs.

Chen *et al.* (2023) investigated the first pan-genome of 516 broomcorn millet to identify the genetic basis of agronomic traits such as nutrient content, salt, drought tolerance, disease, and pest resistance.

Lu *et al.* (2024) provided large-scale genomic variation in broomcorn millet. In total, 1904 accessions were sequenced. Genome-wide association studies (GWAS) pinpointed candidate genes for 12 agronomic traits. For example, accessions with higher expression of PmGW8 demonstrated larger grain size, and the expression of PmLG1 showed loose panicles.

Kuijer *et al.* (2024) determined Striga susceptibility in two contrasting lines of pear millet, Aw and P10. The presence of CLAMT1b in P10 made it more susceptible to Striga, and its absence in the Aw accession demonstrated higher resistance. This gene can play an important role in pearl millet lines and can be useful in marker-assisted breeding and genome editing.

Zhang *et al.* (2024) reconstructed a *Poaceae* phylogeny with nuclear genes from genomic/transcriptomic datasets of >360 grasses. This provided an excellent opportunity to investigate whole-genome duplication (WGD) in multiple subfamilies to identify potential WGDs across *Poaceae*. For example, a WGD identified as the *rho* gene is shared among the *Poaceae* members.

Li *et al.* (2022) provided multiomics datasets encompassing the genomes, transcriptomes, metabolomes, and anti-inflammatory indices from 398 foxtail millet accessions. In total, 83 metabolites were identified in the millet grains with anti-inflammatory effects, such as naringenin chalcone (wh1094) and 5-O-*p*-coumaroylquinic acid (wh1526). The function of the PHYTOENE SYNTHASE 1 (PSY1) gene in affecting millet grain color and quality was validated using CRISPR-mediated genome editing.

Bandyopadhyay *et al.* (2024) evaluated the physiological and genetic basis of nitrogen responsiveness in foxtail millet (*Setaria italica* L.). The study provides insight into the functioning of contrasting NRp and NNRp accessions at the whole plant and flag leaf levels. Understanding the drivers of biomass and N allocation changes at an earlier developmental stage would provide additional useful information on the molecular basis for N responsiveness in *S. italica*, which is of wider relevance for developing more N-responsive cereal crops.

Jaiswal *et al.* (2023) evaluated 104 foxtail millet accessions for 11 nutrients in three different environments, and 67 high-confidence marker–trait associations (MTAs) were identified. Six SNPs showed a pleiotropic effect and were associated with two or more nutrients, whereas 24 candidate genes were identified for 28 MTAs involving seven traits.

Kumar *et al.* (2024) explored the genome-wide distribution of H3K9ac and gene expression in dehydration-tolerant ‘IC 403579’ (IC4) and sensitive ‘IC-480117’ (IC41) cultivars of foxtail millet in response to dehydration stress, emphasizing the functional role of SiHDA9.

Ramu *et al.* (2023) provided *de novo* genome assemblies with high-quality annotation using high-coverage long- and short-read data for three pearl millet genotypes. Gene annotation and enrichment analyses revealed that the millet genome is enriched with cysteine- and methionine-coding genes.

Pangenome sequencing significantly improves the unraveling of the large genetic diversity within a species compared with long-read sequencing. It can also capture genomic diversity from multiple representative genomes, especially with the advent of variation graphs and Giraffe tools that further promote the use of the graph-based pangenome (Raza *et al.*, 2023). Furthermore, it can rediscover lost diversity during domestication and reintroduce selected variation associated with valuable traits. Selective sweep regions were found in the domesticated relative to wild pearl millet accessions, containing structural variants that overlap with genes of improved cultivars that might have contributed to heat adaptation during domestication (Yan *et al.*, 2023). Additionally, utilizing crop wild relatives for the identification of novel genes to improve the allelic richness of the cultivated germplasm can be exploited by the genomic tools for mapping precise traits and introgressions (Rocha *et al.*, 2021). Moving forward, a super-pangenome strategy that extends to the inclusion of accessions beyond the species level (Khan *et al.*, 2020) can deliver a more comprehensive list of genetic variations that have not yet been been applied to millets and can enhance the power of pangenome studies to understand better evolutionary selection sweeps for the reintroduction of advantageous variants in breeding practices.

Molecular markers, genetic linkage maps, and other sequencing information are vital for developing genome-assisted breeding. In pearl millet, one of the first DNA markers to be used for genetic mapping were restriction fragment length polymorphism (RFLP) markers, resulting in the generation of the shortest linkage map (Liu *et al.*, 1994). Various other efforts followed using amplified fragment length polymorphism (AFLP) markers, genomic simple sequence repeats (gSSRs), diversity array technology (DarT), and quantitative trait locus (QTL) mapping. Later, thousands of single nucleotiode polymorphism (SNP) markers were identified using genotyping-by-sequencing (GBS) in global germplasm collections of pearl millet lines (Serba *et al.*, 2019; Kanfany *et al.*, 2020). Improved denser linkage maps with higher coverage were created, making saturated genetic maps available and thousands of SNPs facilitating genetic studies across multiple millet lines (Kumar *et al.*, 2018).

Densely connected genetic linkage maps are used in association mapping studies such as genome-wide association studies (GWAS), genetic selection, or QTL mapping, which are associated with various stress tolerance traits. GWAS in pearl millet identified genetic factors responsible for the variations in flowering time at the phytochrome C (PHYC) (866 bp) locus (Saïdou *et al.*, 2014), significant association with *PgMADS11* alleles (Mariac *et al.*, 2011), as well as the PgPb11603 DArT marker (Gemenet *et al.*, 2015). In addition, SNPs associated with acetyl-CoA carboxylase genes were found to be linked to panicle and grain yield (Sehgal *et al.*, 2015), such as the PgPb12954 and Xibmsp11/AP6.1 markers (Gemenet *et al.*, 2015). Similarly, key alleles were found for grain iron and zinc (Anuradha *et al.*, 2017). Later, a total of 188 inbred lines were evaluated under drought for identifying QTLs associated with agronomic traits using GBS (Debieu *et al.*, 2018), revealing two SNPs to be significantly associated with biomass production. Finally, multienvironmental QTL analysis was carried out on 210 recombinant inbred lines in three diverse pearl millet growing regions in India for three consecutive years. Stable QTLs were identified across environments associated with thousand seed weights and yields per plant (Singhal *et al.*, 2022).

## Next-generation genomics and machine learning tools for millet improvement

Next-generation genomic tools for improving small millets could be part of the ‘Next Green Revolution’—a term used to coin novel methods for crop improvement (Weckwerth *et al.*, 2020). Classical breeding strategies such as mutation and transgenic breeding are laborious and pose many challenges. New breeding techniques such as genetic editing, epigenetic modifications, and heritable targeted mutations can speed up the breeding process and make it more efficient. Techniques such as CRIPSR/Cas9 [clustered regularly interspaced palindromic repeats (CRISPR)/CRISPR-associated protein 9] and genome-wide editing such as virus-induced gene silencing (VIGS), the super-pangenome approach, generation of double haploids, resequencing, and transformation methods such as the nanoparticle-based delivery system (Choudhary *et al.*, 2023) or the *Agrobacterium*-based system (Antony Ceasar and Ignacimuthu, 2011; Satish *et al.*, 2017), will all aid in acheiving this end. More advanced sequencing approaches could be used for complete genome sequencing of several small millets, including kodo millet, Job’s tears, and little millet, to increase the genetic pool for mining of genomic markers as well as the development of C_4_ model systems with shorter generation times, such as *Xiaomi* (foxtail millet), to speed up breeding (Yang *et al.*, 2020).

Novel machine learning-assisted phenomics approaches could accelerate genetic diversity information. With the advent of more omics analyses, big data from transcriptomics, proteomics, and metabolomics require novel tools for useful data mining and retrieval of mechanisms regulating various agronomic traits. Conventional statistical methods fail to capture and model the complex non-linear relationships between molecular and phenomic datasets (Niazian and Niedbała, 2020). Machine learning, as a subset of artificial intelligence (AI), can be used to train neural networks to learn these non-deterministic, highly non-linear associations and to predict high-yielding traits. In the process, features of high predictive accuracy are extracted from omics data and can be used as markers for genomic editing for breeding purposes.

Various deep-learning methods have been applied for genomic selection in several crops (Montesinos-López *et al.*, 2021). In millets, artificial neural networks (ANNs) have been used for production forecasting of pearl millet in Karnataka, India (Vijay and Mishra, 2018). Deep learning convolutional neural networks have been used to detect mildew resistance in pearl millet (Coulibaly *et al.*, 2019). A quality testing system, MCSCQT, was used to classify diseased and normal pearl millet seeds (Kundu *et al.*, 2022). Furthermore, another ANN algorithm was developed to input soil and climatic parameters to aid farmers in selecting the best crop to grow in the current cropping season (Madhuri and Indiramma, 2021). With the recent advent of machine learning tools, data-driven methods, and ANNs, research in small millet is still lacking. Therefore, it presents a major opportunity for progress and integration into successful next-generation breeding strategies.

## Integrated multi-omics and artificial intelligence are crucial for millet improvement - a PANOMICS framework

While the genome and transcriptome layers are rich sources of information for making causal inferences on how genetic architecture influences a phenotypic response, these would not be complete without functional information from the proteome and metabolome layers. We recently introduced the concept of panomics which combines natural genetic variation of large germplasm collections with multiomics analysis, machine and deep learning techniques to exploit the large intraspecific phenotypic and molecular variation for the identification of novel breeding targets (Weckwerth *et al.* 2020). This concept goes beyond classical pangenomics by integrating not only genomic information of genotype panels but also RNAseq, metabolomics, proteomics and phenomics (Weckwerth *et al.*, 2020; Mishra *et al.*, 2024). Proteome and metabolome refer to the whole set of proteins and metabolites, respectively, measured via LC-MS and GC-MS techniques (Weckwerth *et al.*, 2020; Chaturvedi *et al.*, 2024b). Together, integrating big omics data using advanced bioinformatics tools has revolutionized the mechanistic understanding and in-depth knowledge of gene function, pathways, and regulation of stress responses in many underutilized crop species (Muthamilarasan *et al.*, 2019). This holistic multi-omics integrative approach has not shown significant advances for millets, with cases limited to proteomics and metabolomics studies for some species (Singh *et al*., 2021). One of the first comparative proteomic studies in foxtail millet identified 29 proteins differentially expressed under salt stress (Veeranagamallaiah *et al.*, 2008). Later, the shotgun proteomics approach quantified 2281 proteins under drought, showing significant changes within leaf tissue compared with roots and seeds (Ghatak *et al.*, 2016). MALDI-TOF/TOF (matrix-assisted laser desorption ionization-tandem time of flight)-MS analysis of finger millet (Sen *et al.*, 2016) identified 21 jasmonate-interacting proteins, extending the understanding of jasmonate signaling during stress. Furthermore, a tissue-specific proteome analysis identified a stay-green proteome signature in pearl millet genotypes not identified in wheat (Ghatak *et al.*, 2021). This highlighted the superiority of a neglected crop compared with conventional major crops, and revealed proteome signatures of drought tolerance, which are not predictable from genome sequencing alone.

Metabolomics studies in millets have seen a rise in recent years (Pan *et al.*, 2020; Ghatak *et al.*, 2022), establishing millets as great sources of phenolic compounds, flavonoids, and antioxidants. A targeted metabolomics analysis identified 330 annotated metabolites (Wei *et al.*, 2021) in 150 foxtail millet genotypes. Using an LC-MS/MS approach, 2082 differential metabolic signals were detected which are essential for drought tolerance in proso millet and related to energy metabolism, photosynthesis, and anthocyanin production (Cao *et al.*, 2022). Similarly, 627 metabolic signals were detected and linked to grain color in proso millet (Li *et al.*, 2021). Stage-specific metabolic properties were elucidated through the finding of the regulation of 2014 metabolic signals during the grain-filling process in foxtail millet (Wang *et al.*, 2023).

The integration of multi-omics datasets to derive useful clusters from networks or features related to a phenotypic outcome has yet to be highly explored in millets. A study integrated transcriptomics and metabolomics during seed germination of foxtail millet (Pan *et al.*, 2020) and identified flavonoid, lignin, and phenylpropanoid pathways enabling salinity tolerance. The rise of AI-assisted omics techniques has experienced evident advances within the field of plant defense (Murmu *et al.*, 2024). Supervised and unsupervised machine learning algorithms, as well as deep learning architectures have been shown to be superior and can handle high dimensional datasets such as big omics data while being able to discern and prioritize the most relevant features and excel in prediction and classification tasks (Murmu *et al.*, 2024). We have recently developed a novel tool box for the transition of genomic prediction to panomic prediction integrating various levels of omics data such as genome and metabolome data (Schwarzerova *et al.*, 2024). This toolbox integrates different machine learning and deep learning methods to improved prediction accuracy for phenotypic traits and is the basic approach for future panomics applications.The highly exploratory nature of AI models can uncover hidden patterns and associations in the data that are crucial in omics research. Within crop abiotic stress tolerance research, various machine learning methods have achieved >70% accuracy in identifying important abiotic stress genes (Meher *et al.*, 2022). A random forest model was used to predict plant–pathogen protein–protein interactions incorporating sequence data within a network setting (Yang *et al.*, 2019). *Arabidopsis thaliana* mutant lines were classified under control and stress conditions after training a machine learning algorithm using quantified changes in metabolome and proteome data (Fürtauer *et al.*, 2018). This approach successfully identified 23 proteins highly regulated under heat and high-light stress conditions. In another recent study the metabolome of 241 *A. thaliana* lines was investigated with respect to its natural variation and prediction capacity of evolutionary cold stress adaptation processes (Weiszmann *et al.*, 2023). Machine learning and genomic prediction algorithms identified sets of metabolic markers involved in these adaptation processes. An AI based algorithm identified fumarate metabolism as a checkpoint of evolutionary cold stress adaptation in *A. thaliana*.

Another innovation, ‘evolutionarily informed machine learning’ trained on *A. thaliana* transcriptomic data, predicted NUE-related genes in maize (Cheng *et al.*, 2021), showcasing the power of transfer learning from model plant organisms to other crop systems. Transfer learning could also be applied to millets, where the application of new AI tools remains to be explored and has plenty of opportunity for innovation. Multi-omics databases for millets, such as MDSi (Li *et al.*, 2023) or Milletdb (Sun *et al*., 2023), provide readily available resources for immediate exploitation by AI tools to speed up advance predictions related to agriculturally useful traits. A limitation of such large-scale AI tools is their black-box nature, where the underlying model of the data is not known previously and may result in overfitting due to the many unknown variables and parameters.

A solution would be to use data-driven mathematical tools that are hypothesis-driven (i.e. assume basic properties and dynamic trajectories in the data) (Weckwerth, 2019). In this direction, the Jacobian matrix has been used to model linear dynamics in molecular systems (Sun and Weckwerth, 2012; Li *et al.*, 2023; Weiszmann *et al*., 2023; Chaturvedi *et al.*, 2024a). The differential Jacobian matrix can discern differential fluxes which targets for control of abiotic tolerance traits (Nägele *et al.*, 2014) and has recently been applied on chickpea (Chaturvedi *et al.*, 2024a) to elucidate drought responses on yield at three different time points. Data-driven tools are easily applied methods that are only waiting to be used to enhance the value and potential of millets within agriculture. They do not require an explicit model, making them suitable for use in biological systems where model parameters are unknown. They can be easily integrated with neural networks for feature prediction and network integration with improved accuracy.

## Designing millets for precision breeding

Genome editing has emerged as a useful toolbox for plant breeders to improve crop yield by targeting specific functional genes identified through sequencing and structural genomic dissection efforts. Techniques include zinc finger nucleases (ZFNs), transcription activator-like effector nucleases (TALENs), and the most popular, the CRISPR/Cas system (Weckwerth *et al*., 2020). However, high-resolution functional genomic studies involving these advanced tools need complete and fully annotated genome sequences to precisely target the genes and reduce off-target effects (Ceasar, 2022).

Genetic editing has been implemented in many crops, reported mostly in rice, followed by wheat, maize, and barley (Ceasar, 2022), but limited in millets, possibly due to a lack of complete and annotated genome sequence or funding and less market consumption. The first genome editing study using CRISPR/Cas9 was reported in foxtail millet (Lin *et al.*, 2018) due to the availability of high resolution of the fully annotated genome. More recently, single- and multi-gene knockouts for base editing of *SiALS* and *SiACC* genes in foxtail millet (Liang *et al.*, 2021) resulted in the production of a homozygous herbicide-tolerant mutant plant with no off-target effects. Further, the function of the PHYTOENE SYNTHASE 1 (PSY1) gene was validated in its effect on grain color and quality in foxtail millet after a multi-omics association analysis of genetic variants (Li *et al.*, 2022). Functionally evaluated genes belonging to abscisc acid signaling, transcription factorsand signaling gene families were verified for their contribution to drought tolerance mechanisms in pearl millet (Chakraborty *et al.*, 2022). Various *SiLBD* genes were found to be involved in abiotic stresses in foxtail millet and functionally validated by ectopic expression in Arabidopsis and rice (Li *et al.*, 2023). Functional characterization of *AtROXY19* (glutaredoxin-encoding gene homolog) revealed a novel role for this glutaredoxin gene clade in regulating cell elongation (de la Fuente Cantó *et al.*, 2024). In addition, foxtail mosaic virus (FoMV)-based gene silencing was first reported for foxtail millet as a VIGS system for high-throughput functional genomics in monocots that resulted in silencing of phytoene desaturase and magnesium chelatase genes (Liu *et al.*, 2016). Even higher editing efficiency was observed for FoMV-targeted SvCA2 of millet, with 60% in systemic leaves (Mei *et al.*, 2019). To enhance reverse genetic studies in millets, efficient transformation and regeneration protocols are essential, potentially replacing the need for transforming millets with CRISPR and other gene constructs. Genes of different millets that have been functionally characterized for their role in abiotic stress and metal toxicity are summarized in Box 3.

Box 3. **Functionally characterized genes from major and minor millets in response to abiotic stress and metal toxicity (based on research performed in the last 4 years)**

**Sorghum bicolor**

*SbYS1* and *SbWRKY72*: transgenic Arabidopsis overexpressing *SbYS1* accumulated less cadmium, while overexpression of *SbWRKY72* led to cadmium sensitivity (Jia *et al.*, 2024).

*SbMYC2*: overexpression enhanced drought tolerance in Arabidopsis, rice, and sorghum (Wang *et al.*, 2024).

*SbMYBHv33*: overexpression reduced biomass accumulation and salinity tolerance in transgenic sorghum (Zheng *et al.*, 2023).

*SbEXPA11*: overexpression in transgenic sorghum led to enhanced photosynthesis and tolerance to cadmium stress (Wang *et al.*, 2023).

*miR-6225-5p*: overexpression in sorghum inhibited plant growth under salt stress (Sun *et al.*, 2023).

*SbNAC9*: overexpression in sorghum seedlings led to enhanced drought tolerance while silencing resulted in drought sensitivity (Jin *et al.*, 2023).

*SbWRKY55*: negatively regulated salinity stress responses in transgenic sorghum and Arabidopsis (Song *et al.*, 2022).

*SbHDT701*: overexpression in *Escherichia coli* resulted in increased acetylation modification levels and provided tolerance to salinity and dehydration (Du *et al.*, 2021).

*SbMATE*: transgenic sugarcane overexpression led to enhanced aluminum tolerance (Ribeiro *et al.*, 2021).

**Pearl millet**

*PgWRKY74*: overexpression in Arabidopsis resulted in delayed shoot growth under dehydration and salinity stress (Qazi *et al.*, 2024).

*PgP5CS*: expression in transgenic tobacco conferred improved abiotic stress tolerance (Sellamuthu *et al.*, 2024).

*PgRWP-RK*: overexpression led to enhanced heat tolerance in rice (Yan *et al.*, 2023).

*PgDREB2A*: Arabidopsis expression resulted in enhanced heat, drought, and salinity tolerance (Meena *et al.*, 2022).

*PgGPx*: overexpression in transgenic rice led to enhanced tolerance to Cd^2+^ stress (Islam and Reddy, 2022).

*PgPIP2;6*: overexpression improved drought and heat stress tolerance (Reddy *et al.*, 2022).

*PgLEAPC*: transgenic tobacco overexpressing this gene displayed improved tolerance to various abiotic stresses (Divya *et al.*, 2021).

**Foxtail millet**

*SiCYP19-a* and *SiCYP19-b*: *o*verexpression conferred salt tolerance in foxtail millet (Zhang *et al.*, 2024).

*SiHDA9*: virus-induced gene silencing (VIGS) of *SiHDA9* conferred dehydration tolerance to foxtail millet (Kumar *et al.*, 2024).

*SiSnRK2.6*: overexpression in foxtail millet enhanced the resistance to low potassium stress (Ma *et al.*, 2024).

*SiMYB30*: conferred tolerance to low nitrogen stress in transgenic rice (Zhang *et al.*, 2023a).

*SiATG8a*: overexpression in transgenic wheat conferred tolerance to phosphorus starvation (He *et al.*, 2023b).

*SiGRF1*: overexpression in foxtail millet led to reduced sensitivity to drought stress (Zhang *et al.*, 2023b).

*SiDi19-3*: *o*verexpression provided salt tolerance to transgenic foxtail millet and Arabidopsis (Xiao *et al.*, 2023).

*SisHSP21.9*: transgenic rice overexpression resulted in improved tolerance to high temperature stress (Singh *et al.*, 2022).

*SiMYB19*: expression in transgenic rice provided tolerance to salinity stress (Xu *et al.*, 2022).

*SiNRX1*: overexpression in Arabidopsis resulted in tolerance to drought and salt stress (Zhang *et al.*, 2022).

**Finger millet**

*EcCAX3*: overexpression conferred tolerance to metal and ions stress in yeast and Arabidopsis (Jamra *et al.*, 2024).

*EcDREB2A*: overexpression in tobacco enhanced tolerance to heat stress through ROS scavenging (Singh *et al.*, 2021).

*EcCaM*: transgenic Arabidopsis expression conferred tolerance to drought and salinity (Jamra *et al.*, 2021).

**Broomcorn millet**

*PmABI5*: overexpression in Arabidopsis enhanced abiotic stress (An *et al.*, 2022).

**Tef**

*EtSD-1*: CRISPR/Cas9-mediated mutation of *EtSD-1* improved lodging resistance in tef (Beyene *et al.*, 2022).

## Future perspectives

**Fig. 2.**
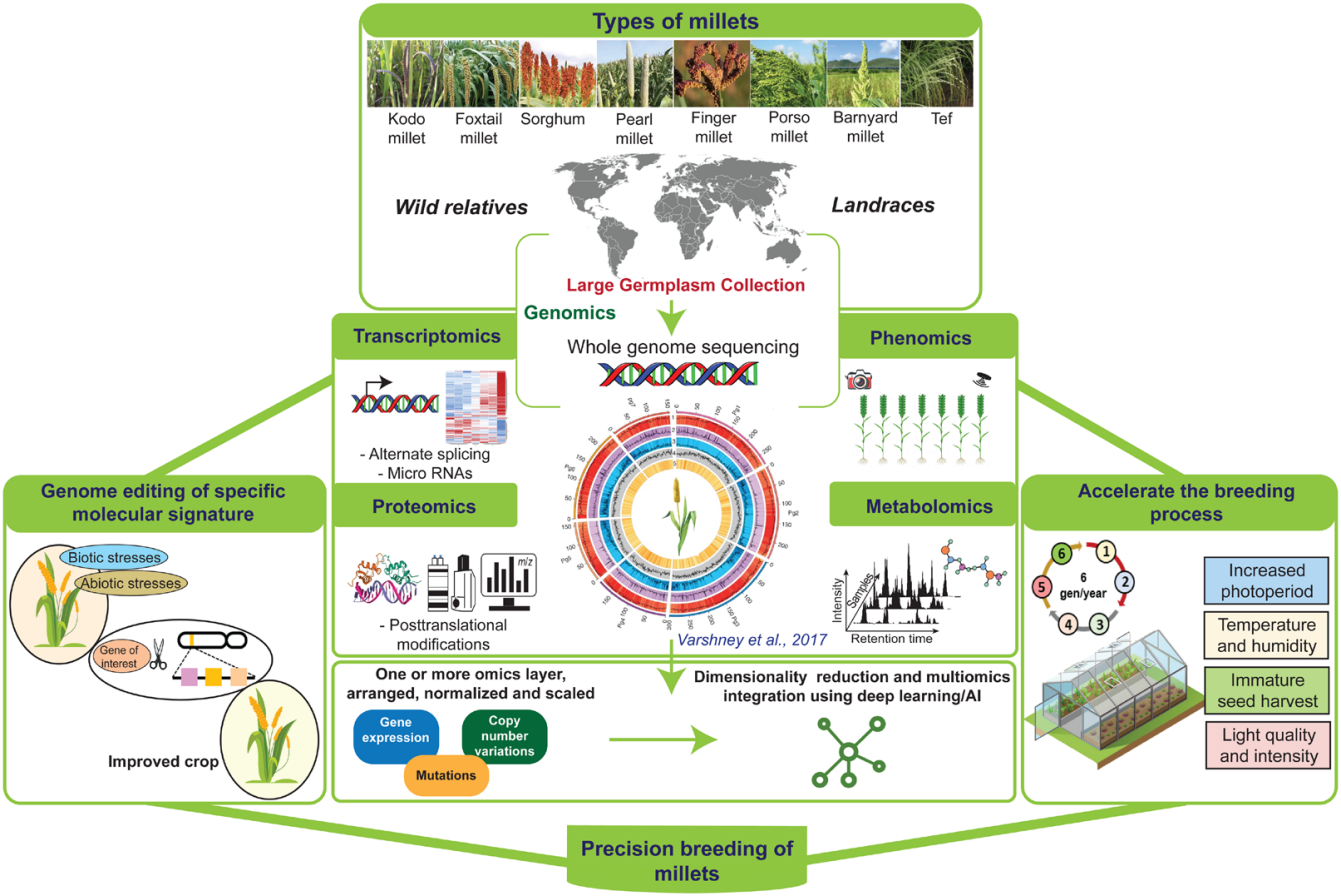
Comprehensive PANOMICS and system driven approach for future breeding programs focused on millet improvement and precision breeding. AI, artificial intelligence.

Millets were once considered orphan crops due to the low amount of attention they received during the Green Revolution. However, they are now receiving more attention in the modern genetic era. The lack of complete and annotated genome sequences and efficient regeneration and transformation systems may hinder high-resolution millet research. Therefore, it is crucial to explore the genetic resources of millet further to identify key molecular markers for trait association mapping. This can lead to marker-associated improvement in millet, and similar markers can also be studied in other related crops. Additionally, applying an advanced PANOMICS approach combined with advanced AI tools can help unravel natural genetic variation and enable the functional interpretation and characterization of genes, opening up new opportunities for millet improvement (Fig. 2). Furthermore, using the genome editing (CRISPR/Cas) toolkit can facilitate sequence-specific targeted genome editing to achieve desired traits in the genotype, which could revolutionize millet breeding. The progress in millet research and the information generated can play a crucial role in achieving the sustainable development goal of the UN, which is to promote sustainable agriculture, end hunger, achieve food security, and improve nutrition.

## Data Availability

This review contains no new experimental data.
